# Impact of upper airway abnormalities on the success and adherence to mandibular advancement device treatment in patients with Obstructive Sleep Apnea Syndrome^☆^^☆☆^

**DOI:** 10.1016/j.bjorl.2015.08.005

**Published:** 2015-09-07

**Authors:** Renato Prescinotto, Fernanda Louise Martinho Haddad, Ilana Fukuchi, Luiz Carlos Gregório, Paulo Afonso Cunali, Sérgio Tufik, Lia Rita Azeredo Bittencourt

**Affiliations:** a Department of Psychobiology, Universidade Federal de São Paulo (UNIFESP), São Paulo, SP, Brazil; b Department of Otorhinolaryngology, Faculdade de Medicina do ABC, Santo André, SP, Brazil; c Dentistry Course, Universidade Federal do Paraná (UFPR), Curitiba, PR, Brazil; d Department of Medicine and Sleep Biology, Universidade Federal de São Paulo (UNIFESP), São Paulo, SP, Brazil

**Keywords:** Obstructive sleep apnea, Removable orthodontic appliances, Physical examination, Nose, Apneia do sono tipo obstrutiva, Aparelhos ortodônticos removíveis, Exame físico, Nariz

## Abstract

**Introduction:**

The mandibular advancement device (MAD) is a option to treat patients with Obstructive Sleep Apnea Syndrome (OSAS).

**Objective:**

To assess the influence of upper airway abnormalities on the success of and adherence to MAD in patients with OSAS.

**Methods:**

Prospective study with 30 patients with mild to moderate OSAS and indications for MAD. The protocol included questionnaires addressing sleep and nasal complaints, polysomnography, and upper airway assessment. The analyzed parameters of patients who showed therapeutic success and failure and those who exhibited good and poor treatment adherence were compared.

**Results:**

28 patients completed the protocol; 64.3% responded successfully to treatment with MAD, and 60.7% exhibited good adherence to treatment. Factors associated with greater success rates were younger age (*p* = 0.02), smaller cervical circumference (*p* = 0.05), and lower AHI at baseline (*p* = 0.05). There was a predominance of patients without nasal abnormalities among patients treated successfully compared to those with treatment failure (*p* = 0.04), which was not observed in relation to adherence. Neither pharyngeal nor facial skeletal abnormalities were significantly associated with either therapeutic success or adherence.

**Conclusion:**

MAD treatment success was significantly lower among patients with nasal abnormalities; however, treatment adherence was not influenced by the presence of upper airway or facial skeletal abnormalities.

## Resumo

**Introdução:**

O Aparelho Intraoral (AIO) é uma opção para tratamento da Síndrome da Apneia Obstrutiva do Sono (SAOS).

**Objetivos:**

Avaliar a influência das alterações da VAS e esqueléticas faciais através de uma avaliação clinica sistematizada no sucesso e adesão ao (AIO) em pacientes com (SAOS).

**Método:**

Estudo prospectivo em que foram avaliados 30 pacientes com SAOS leve a moderada e indicação de AIO. Protocolo incluiu questionários de sono e queixas nasais; polissonografia e avaliação da VAS por rinoscopia anterior e oroscopia. Os parâmetros analisados foram comparados entre pacientes com sucesso e insucesso, e com boa e má adesão à terapia.

**Resultados:**

Completaram o protocolo 28 pacientes. O sucesso ao tratamento foi de 64,3% e a adesão 60,7%. Os fatores associados ao sucesso foram menor idade, menor circunferência cervical e menor IAH basal. Quanto à presença de alterações nasais, houve predomínio de pacientes sem alteração nasal entre os pacientes com sucesso comparados àqueles com insucesso (p = 0,04); o que não foi observado em relação à adesão. Quanto às alterações faríngeas e alterações esqueléticas faciais, não houve significância.

**Conclusão:**

O sucesso do tratamento com AIO foi significativamente menor nos pacientes com alterações nasais, porém a adesão não foi influenciada pela presença de alterações de VAS ou esqueléticas faciais.

## Introduction

The treatment of choice for Obstructive Sleep Apnea Syndrome (OSAS) is the use of continuous positive airway pressure (CPAP), especially in severe cases.^1^ In mild to moderate and primary snoring cases, other treatments can be used, such as the mandibular advancement device (MAD).^1^, ^2^

It is estimated that nasal obstruction is present in approximately 64% of patients with OSAS and most of these patients have associated anatomical alterations, such as deviated septum and inferior turbinate hypertrophy.^3^ Although there have been studies demonstrating the presence or absence of an association between nasal alterations and their treatment with CPAP adherence,^3^, ^4^, ^5^ no studies have demonstrated whether the nasal and upper airway (UA) alterations might or might not interfere with successful treatment by or adherence to MAD.

Zeng et al. obtained data suggesting that increased nasal resistance can negatively influence MAD treatment outcomes; it represents, to date, the only study in literature that performed nasal assessment through rhinomanometry in patients with MAD.^6^

The scarcity of studies that have assessed the presence of UA and facial skeleton alterations using otorhinolaryngological physical examination in patients with OSAS referred for treatment with MAD, and have also evaluated the association of these alterations with treatment success and adherence, were the reasons that prompted this research. Thus, this study aimed to evaluate the influence of UA and facial skeletal alterations through a systematic and standardized clinical assessment of the success and adherence to treatment of OSAS with MAD.

## Methods

### Sample

A total of 30 adult patients from the outpatient clinic specialized in treating sleep-related disorders during 2006 and 2007, who had polysomnography-confirmed mild to moderate OSAS according to the diagnostic criteria of the International Classification of Sleep Disorders, 2005,^7^ with indication for MAD were included in this study. The protocol was submitted to the ethics and research committee, CEP No. 0162/06. Patients who agreed to participate signed the informed consent.

Inclusion criteria were patients aged 25–65 years, of both genders, with mild to moderate OSAS, with apnea–hypopnea index (AHI) between 5/h and 30/h.

Exclusion criteria included patients with other sleep disorders rather than OSAS, with previous clinical or surgical treatments for OSAS, users of alcohol, stimulants, or sedatives, those with loss of posterior dental support that would compromise the retention of MAD, those with active periodontal disease, and those with protrusion displacement <6 mm.

### Protocol

All patients were submitted to an evaluation protocol, which consisted of applying a sleepiness questionnaire (Epworth Sleepiness Scale [ESS]),^8^ anthropometric examination (neck circumference and body mass index [BMI]), UA and facial skeletal examination, and overnight polysomnography with MAD. The protocol was applied on the day the MAD was provided and after 120 days of MAD use.

Patients also completed a daily questionnaire on MAD use and a sleep diary, which were completed during all 120 days of the study.

After the end of the protocol, it was possible to separate patients between groups with good and poor compliance, and between groups with treatment success and treatment failure with MAD. The clinical data, polysomnographic data, and upper airway and facial skeleton physical examination findings were compared between the groups. The criteria used for treatment success and adherence are described below.

### Tools used in the study

All dental assessment and follow-up of patients were carried out by the same dentist, trained in the area of sleep medicine. Before the study, all patients were submitted to orthodontic assessment and the maximum mandibular advancement was measured. Next, the MAD was custom-made for each patient, with an initial mandibular advancement of 50% of the maximum mandibular advancement. After the start of the protocol, patients were instructed to return weekly for the MAD to be advanced by 0.5 mm at each visit, until they reached the maximum comfortable mandibular advancement. During these return visits, patients brought the sleep and MAD use diaries, which will be further detailed below. Any eventual complications were treated.

The MAD model used in the study was the Brazilian Dental Appliance (BRD). BRD is an adjustable mandibular repositioning device. It has two independent expanding mechanisms – screws positioned with the long axis in the anteroposterior direction. Two independent palate rods emerge from these expanding mechanisms, one on the right and one on the left, which are inserted inferiorly into two small tubes located in the anterior portion, distal to the mandibular canines, of the lower acrylic support base. This proposed design allows successive advances in the mandibular position, without preventing lateral mandibular movements. In other words, when the device is in place, even when the jaw is in a more anterior position, patients may perform lateral movements and have a small mouth opening.^9^

### Questionnaires

The patients were assessed for daytime sleepiness by applying the ESS.^8^ The scale was administered to both groups, both at the baseline and at the end of the assessment. The sum of the total score ranges from 0 (zero) to 24, and scores >9 suggest excessive daytime sleepiness.

The sleep and MAD-use dairies consisted of notation tables, whose purpose was to provide the necessary information for the study and that contained the days of the month and the week. In them, the patients recorded a series of information during the study phases, including the time their sleep started and finished, when they awakened during the night, and when they put on or removed the device.

The sleep and MAD-use diaries were used to define whether patients had good or poor adherence to MAD, with the former being those who had used it for more than 90% of the time in the previous week.

### Upper airway examination^3^, ^10^

Upper airway (UA) physical examination consisted of anterior rhinoscopy, oroscopy, and flexible fiberoptic nasal laryngoscopy. Patients considered as having nasal alterations were those with septal deviation grade II (deviation that compresses the inferior nasal turbinate) or III (deviation that compresses the inferior nasal turbinate, touching the lateral wall), or those with grade I septal deviation (deviation that does not touch the inferior nasal turbinate) associated with frequent nasal obstruction complaints (present every day or almost every day) or complaints of frequent rhinitis (rhinorrhea and/or sneezing and/or pruritus, every day or almost every day), or those with inferior turbinate hypertrophy on physical examination.

Those with pharyngeal abnormalities had three or more of the following alterations: soft palate webbing, posterior soft palate, thick soft palate, long uvula, and thick uvula.

The variables modified Mallampati classification and size of palatine tonsils was studied separately, with grade III and IV tonsils (occupying more than 50% of the oropharyngeal space) considered hyperplastic.

In addition, patients with presence or absence of facial skeletal alterations were grouped, considered as present when one or more of the following alterations were observed: retrognathia at facial profile assessment, ogival hard palate, or Angle class II dental occlusion.

### Polysomnography with MAD

Overnight polysomnography with MAD was performed by previously trained professionals, after 120 days of use. The polysomnography device used was an EMBLA computerized system (EMBLA™ S7000; EMBLA Systems, Inc. – Broomfield, CO, United States). The biological variables were measured by electroencephalography (C3/A2, C4/A1, O1/A2, O2/A1), bilateral electro-oculography, submental and tibial electromyography, electrocardiography (modified V2), oral and nasal breathing through a thermistor and nasal flow through a cannula with pressure transducer, thoracic and abdominal movements by uncalibrated inductance plethysmography, snoring by a microphone, oxyhemoglobin saturation by pulse oximetry, and body position sensor.

Sleep staging was performed by a trained polysomnographist, based on the criteria of Rechtschataffen and Kales.^11^ Respiratory and arousal events, as well as sporadic lower-limb movements, were analyzed according to criteria established by the American Academy of Sleep Medicine.^12^

Polysomnography was also used to define which patients achieved treatment success, as a 50% AHI reduction and ≤10 events/h defined this criterion.

### Statistical analysis

The Kolmogorov–Smirnov normality test was applied for all variables, which were expressed as mean and standard deviation, as they showed normal distribution.

Student's *t*-test was used for comparison between the groups for independent samples and GLM (two-way repeated measures ANOVA). Student's *t*-test for dependent samples was used for comparisons before and after treatment in all patients.

The chi-squared test was used to compare categorical variables. A logistic regression model was also used, with treatment success and adherence used as dependent variables. The significance value was set at 0.05 and the software used was Statistic 6.1.

## Results

Of the 30 selected patients, two did not return for the assessment and, therefore, 28 completed the protocol and were included in the study. No patients stopped using the MAD during the protocol.

As for the assessed descriptive data, the mean age of patients was 48.8 ± 11.3 years; nine (32.1%) were males and 19 (67.9%) were females. The mean BMI was 27.4 ± 3.8 kg/m^2^, and the mean neck circumference was 38.3 ± 3.3 cm.

After all patients had completed the baseline assessment prior to using MAD and had achieved the maximum comfortable protrusion with MAD, the questionnaires and polysomnographic parameters revealed some significant differences: decrease in the Epworth Sleepiness Scale (ESS) score from 13.4 ± 6.1 to 11.7 ± 6.3 (*t*_26_ = 2.47; *p* = 0.02); reduction of AHI from 17.5 ± 8.8 to 8.8 ± 6.0 (*t*_27_ = 6.2, *p* < 0.001); decreased arousal index, from 15.9 ± 6.7 to 10.1 ± 5.1 (*t*_27_ = 4.5; *p* < 0.001), and decrease in the percentage of time with oxygen saturation <90%, from 0.71 ± 1.4 to 0.07 ± 0.14 (*t*_26_ = 2.5, *p* = 0.017).

Of the 28 patients, 17 (60.7%) had good adherence to MAD use, whereas 11 (39.3%) showed poor adherence. The clinical parameters at baseline showed no statistically significant differences between the good and poor adherence groups (Table 1).

**Table 1:** Comparison of clinical parameters between patients with good and poor adherence to MAD at baseline.

	Good adherence (*n* = 17)	Poor adherence (*n* = 11)	*t*	df	*p*
Age (years)	47.9 ± 10	50.3 ± 12	−0.5	26	0.59
BMI (kg/m^2^)	28.5 ± 3	25.7 ± 3	1.9	26	0.06
NC (cm)	37.1 ± 3	35.1 ± 2	1.6	26	0.10

BMI, body mass index; NC, neck circumference.

Statistically significant *p*-value <0.05 (Student's *t*-test for independent samples).

The AHI values (*F* = 4.5; *p* < 0.05) and arousal index (*F* = 6.9; *p* < 0.05) differed between the groups at baseline, and were higher in the poor adherence group (Table 2).

**Table 2:** Comparison of the Epworth Sleepiness Scale and polysomnography parameters between patients with good and poor adherence to the mandibular advancement device (MAD) at baseline and after 120 days of MAD use.

	Good adherence (*n* = 17)		Poor adherence (*n* = 11)		*p* (group and time interaction)
	Basal	With MAD	Basal	With MAD	
ESS	13.6 ± 6	12.1 ± 6^a^	12.0 ± 6	9.6 ± 6^a^	0.74
AHI (events/hour)	14.7 ± 7	7.5 ± 5^a^	23.2 ± 9^b^	11.3 ± 6^a^	0.18
SE (%)	87.2 ± 5	87.6 ± 5	88.3 ± 9	86.0 ± 9	0.15
N3 (%)	18.1 ± 5	17.7 ± 7	18.2 ± 5	21.2 ± 11	0.32
REM (%)	18.9 ± 3	19.3 ± 4	20.0 ± 6	22.3 ± 6	0.66
AI (events/hour)	13.3 ± 6	9.2 ± 4^a^	20.7 ± 4^b^	11.6 ± 5^a^	0.09
SpO_2_ MIN (%)	86.3 ± 3	87.4 ± 5	85.2 ± 4	88.4 ± 2	0.34
SpO_2_ <90% (%)	0.4 ± 1	0.04 ± 0.1^a^	1.3 ± 1.9	0.1 ± 0.2^a^	0.18

ESS, Epworth Sleepiness Scale; AHI, apnea/hypopnea index per hour of sleep; SE, sleep efficiency; N3, percentage of slow-wave sleep; REM, percentage of rapid eye movement sleep; AI: arousal index per hour of sleep, SpO_2_ MIN: minimum oxyhemoglobin saturation; SpO_2_ <90%, percentage of desaturation time <90%; NS, non-significant statistical value. GLM test for repeated measures (two-way ANOVA).

Statistically significant *p*-value <0.05.

a *p* < 0.05 (baseline × with MAD).

b *p* < 0.05 (good adherence × poor adherence at baseline).

With use of the MAD in both groups (both good and poor adherence) there was a decrease in the ESS score (*F*7.1 = 5.9, *p* < 0.05), AHI (*F* = 41.8; *p* < 0.05), arousal index (*F* = 24.4; *p* < 0.05), as well as in the percentage of desaturation time below 90% (*F* = 8.18; *p* < 0.05; Table 2).

Of the 28 patients, 18 (64.3%) attained treatment success, according to the standard criteria. When comparing the two groups at baseline – successful and unsuccessful treatment – the only clinical parameter that showed a statistically significant difference was a younger age in the treatment success group (44.8 ± 9 *vs*. 56 ± 10; *t* = −2.8; df = 26; *p* < 0.001; Table 3).

**Table 3:** Comparison of clinical parameters between patients showing successful and unsuccessful treatment with MAD at baseline.

	Successful (*n* = 18)	Unsuccessful (*n* = 10)	*t*	df	*p*
Age (years)	44.8 ± 9	56 ± 10	−2.8	26	<0.001
BMI (kg/m^2^)	27.1 ± 4	28 ± 3	−0.6	26	0.54
NC (cm)	36.3 ± 3	36.4 ± 2	−0.07	26	0.94

BMI, body mass index; NC, neck circumference.

Statistically significant *p*-value <0.05 (Student's *t*-test for independent samples).

When comparing the groups with treatment success and failure at baseline, there were no differences in the ESS and PSG parameters, but after treatment with MAD, it was observed that the patients in the success group had lower AHI values (*F* = 14.2; *p* < 0.01) and arousal index (*F* = 7.1; *p* < 0.05), as expected by the adopted success criteria (Table 4).

**Table 4:** Comparison of the Epworth Sleepiness Scale and polysomnography parameters between patients showing successful and unsuccessful treatment with MAD at baseline and 120 days after MAD use.

	Successful (*n* = 18)		Unsuccessful (*n* = 10)		*p* (group and time interaction)
	Basal	With MAD	Basal	With MAD	
ESS	13.8 ± 4	11.9 ± 5^a^	11.8 ± 8	10.0 ± 7^a^	0.95
AHI (events/hour)	15.2 ± 8	5.2 ± 2^a^	22.3 ± 8	15.8 ± 5^a^^,^^b^	0.26
SE (%)	89.0 ± 5	89.3 ± 5	85.0 ± 9	82.8 ± 8	0.34
N3 (%)	17.5 ± 6	20.2 ± 8	19.4 ± 4	16.3 ± 10	0.09
REM (%)	19.4 ± 5	20.7 ± 4	19.1 ± 4	19.6 ± 7	0.71
AI (events/hour)	14.7 ± 6	7.7 ± 3^a^	17.9 ± 6	14.5 ± 5^a^^,^^b^	0.22
SpO_2_ MIN (%)	86.2 ± 3	88.9 ± 2	85.4 ± 3	85.6 ± 6	0.27
SpO_2_ <90% (%)	0.6 ± 1	0.03 ± 0.05^a^	0.9 ± 1	0.1 ± 0.2^a^	0.71

ESS, Epworth Sleepiness Scale; AHI, apnea/hypopnea index per hour of sleep; SE, sleep efficiency; N3, percentage of slow-wave sleep; REM, percentage of REM sleep; AI, arousal index per hour of sleep; SpO_2_ MIN, minimum oxyhemoglobin saturation; SpO_2_ <90%, percentage of desaturation time <90%; NS, non-significant statistical value. GLM test for repeated measures (two-way ANOVA).

Statistically significant *p*-value <0.05.

a *p* < 0.001 (basal × with MAD).

b *p* < 0.05 (treatment success × failure with MAD).

When patients in both groups were assessed together (treatment success and failure), after MAD use, there was a reduction in the ESS (*F* = 5.9; *p* < 0.05); AHI (*F* = 28.2, *p* < 0.001); arousal index per hour of sleep (*F* = 13.0; *p* < 0.01); and the percentage of desaturation time <90% (*F* = 6.0; *p* < 0.05; Table 4).

When assessed individually or in groups, the variables used to form the groups of patients with pharyngeal, nasal, and craniofacial alterations showed no significant differences in relation to treatment adherence (Table 5). As for treatment success, it was observed that the presence of obstructive septal defects (grades II or III; *p* = 0.04) and presence of nasal alterations (*p* = 0.04) were more frequent in the treatment failure group (Table 6); this was not observed for the pharyngeal and craniofacial variables.

**Table 5:** Comparison of upper airway assessment and craniofacial parameters between patients with good and poor adherence to MAD at baseline.

Individual variables	Good adherence (*n* = 17)	Poor adherence (*n* = 11)	*p*
Soft palate webbing	11 (64.7%)	9 (81.8%)	0.30
Posterior soft palate	6 (35.3%)	5 (45.4%)	0.44
Thick soft palate	3 (17.6%)	1 (9%)	0.48
Thick uvula	10 (58.8%)	7 (63.6%)	0.56
Long uvula	10 (58.8%)	5 (45.4%)	0.38
Pharyngeal alteration	9 (52.9%)	6 (54.5%)	0.62
MMI class III/IV	16 (94.1%)	10 (90.9%)	0.64
Grade III/IV palatine tonsils	1 (5.9%)	0 (0.0%)	0.60
Class II dental occlusion	1 (5.9%)	1 (9%)	0.64
Retrognathia	1 (5.9%)	2 (18.2%)	0.34
Craniofacial alteration	11 (64.7%)	6 (54.5%)	0.44
Ogival hard palate	9 (52.9%)	4 (36.4%)	0.32
Frequent nasal obstruction	11 (64.7%)	4 (36.4%)	0.14
Frequent rhinopathy complaint	10 (58.8%)	5 (45.4%)	0.38
Grade I septal deviation	8 (47%)	4 (36.4%)	0.43
Grade II/III septal deviation	3 (17.6%)	2 (18.2%)	0.67
Inferior nasal turbinate hypertrophy	10 (58.8%)	4 (36.4%)	0.22
Nasal alteration	10 (58.8%)	5 (45.5%)	0.38

MMI, modified Mallampati index; *p*, *p*-value (chi-squared test).

**Table 6:** Comparison of upper airway assessment and craniofacial parameters between patients with successful and unsuccessful treatment with MAD at baseline.

Individual variables	Successful (*n* = 18)	Unsuccessful (*n* = 10)	*p*
Soft palate webbing	13 (72.2%)	7 (70%)	0.61
Posterior soft palate	6 (33.3%)	5 (50%)	0.32
Thick soft palate	3 (16.7%)	1 (10%)	0.55
Thick uvula	13 (72.2%)	4 (40%)	0.10
Long uvula	11 (61.1%)	4 (40%)	0.25
Pharyngeal alteration	12 (66.7%)	3 (30%)	0.07
MMI class III/IV	16 (88.9%)	10 (100%)	0.40
Grade III/IV palatine tonsils	1 (5.5%)	0 (0.0%)	0.64
Class II dental occlusion	2 (11.1%)	0 (0%)	0.40
Retrognathia	1 (5.5%)	2 (20%)	0.28
Ogival hard palate	9 (50%)	4 (40%)	0.46
Craniofacial alteration	11 (61.1%)	6 (60%)	0.66
Frequent nasal obstruction	10 (55.5%)	5 (50%)	0.54
Frequent rhinopathy complaint	10 (55.5%)	5 (50%)	0.54
Grade I septal deviation	8 (44.4%)	4 (40%)	0.57
Grade II/III septal deviation	1 (5.5%)	4 (40%)	0.04^a^
Inferior nasal turbinate hypertrophy	7 (38.9%)	7 (70%)	0.12
Nasal alteration	7 (38.9)	8 (80%)	0.04^a^

MMI, modified Mallampati index; *p*, *p*-value (chi-squared test).

a Statistically significant value.

The logistic regression model was used to identify factors associated with treatment adherence and success. The following variables were included: age, gender, neck circumference, ESS, AHI, arousal index, BMI, pharyngeal alterations, nasal alterations, and craniofacial alterations. The findings did not show statistically significant factors associated with adherence; however, regarding treatment success, the younger the age (OR: 0.74 [0.58–0.95]; *p* = 0.02), the smaller the neck circumference (OR: 0.48 [0.23–1.00]; *p* = 0.05) and the lower the baseline AHI (OR: 0.75 [0.57–1.00]; *p* = 0.05), the greater the success (Table 7).

**Table 7:** Logistic regression of factors associated with successful treatment with MAD.

	*B*	*p*	Exp (*B*) − OR	95% CI for Exp (*B*)	
				Minimum	Maximum
Age	−0.298	0.02	0.74	0.58	0.95
NC	−0.730	0.05	0.48	0.23	1.00
AHI pre	−0.285	0.05	0.75	0.57	1.00

NC, neck circumference; AHI, apnea/hypopnea index; OR, odds ratio; CI, confidence interval.

## Discussion

The main finding of this study was that successful treatment with MAD was significantly less likely in patients with nasal alterations; this was not influenced by the presence of other abnormalities. However, there was no influence of UA and facial skeletal alterations on treatment adherence.

When patients were assessed for adherence before and after MAD treatment, it was observed that those with good adherence (60.7%), according to the criteria used, had lower values of AHI and arousals at baseline, suggesting that patients in this group had a milder condition than those in the poor adherence group. Although the studies are contradictory when stating the association between OSAS severity and adherence to MAD,^13^, ^14^ it is known that MAD is more effective in patients with mild OSAS, in whom a normal AHI can be reached more easily and, therefore, where the disease is treated in a more satisfactory way. Thus, in this group of patients with lower AHI values and less sleep fragmentation, sleep consolidation was probably more effective and, consequently, device use was longer-lasting and may have altered the adherence in a positive manner.

The authors did not observe any association between adherence to MAD treatment and any of the assessed anatomical UA or craniofacial alterations although some believe that UA alterations as a result of MAD use could result in some degree of open-mouth breathing at night that could lead to difficulty in maintaining lip occlusion during sleep and cause the patient to remove it, decreasing the device use and adherence, To date, there have been no studies in the literature that made a systematic assessment of UAs and correlated these data with MAD treatment adherence.

Adherence to MAD has been assessed in other studies, which have shown that adherence does not depend on isolated factors, but on a number of them, making patients successful or unsuccessful users of MAD. Marklund et al. found that 24% of their patients stopped using MAD in the first year of treatment, and there was no association with disease severity, age, gender, nasal obstruction, or smoking habits.^14^

Almeida et al. affirmed that adherence can be variable (ranging from 4% to 82% in studies), depending on the type of device employed, disease severity, and possibly patient management.^13^ According to these authors, the most common cause for treatment discontinuation was patient discomfort. The same study stated that most existing studies assess adherence subjectively, and that only one used an adherence monitor, which indicated a mean time of use of 6.8 h per night.^15^

Adherence was evaluated based on the quantification of the time of MAD use, according to information found in patients’ diaries. Although the only available data was that provided by the patient's own account, who could omit or alter the information, this way of obtaining information was the closest to quantitative data concerning adherence.

MAD therapy success was demonstrated with the data obtained in this study in the entire group and after analysis of adherence and success groups, as there was a significant reduction in AHI, arousal index per hour of sleep, percentage of time with oxyhemoglobin saturation <90%, and the ESS score. These data are consistent with the literature, which shows several studies highlighting these findings in patients with mild to moderate OSAS.^2^, ^13^

The success rate was 64.3% according to the criteria we used, a 50% reduction in baseline AHI associated with AHI <10 events/h with the implemented treatment. However, even in the group considered unsuccessful, there was a decrease in ESS score, AHI, and arousal index, as well as improved oxyhemoglobin saturation. Using the same criteria, a reference was found in the literature in which the success rate was 46%.^16^

Other authors have also demonstrated similar rates according to the same criteria.^17^, ^18^ We believe the wide range of criteria used in the literature to characterize MAD treatment success is a major limiting factor for the comparison of further studies with what has already been published. Currently, there is a tendency to consider a third group, that of patients with complete response to treatment, characterized by a reduction in AHI <5 events/h.^19^ In our view this should be done cautiously, since according to current diagnostic criteria for OSAS, an individual is not considered as having this syndrome if he does not show symptoms of snoring, excessive daytime sleepiness, and witnessed apnea, even if the AHI is between 5 and 15 events/h.^7^

Another statistically significant finding was the younger age in the group with successful treatment, confirmed by logistic regression as one of the predictors for the success of the MAD. This was also observed by Almeida et al. and Dal-Fabbro et al.^9^, ^13^

Data regarding treatment success predictors is addressed in the literature. Otsuka et al. observed that success is lower in patients with weight gain and greater anteroposterior upper airway diameter.^20^ It has also been reported that younger patients with positional OSAS (supine), with lower baseline AHI, and with smaller neck circumference have a higher chance of success.^13^, ^21^ Marklund et al. also reported that a successful outcome with positional OSAS is more common in men, whereas the association with mild apnea is more prevalent in women.^14^ The logistic regression observed in this study shows that reduced neck circumference and lower basal AHI, in addition to younger age, can also be considered successful MAD treatment predictors.

Patients were considered as having pharyngeal alterations when they had three or more of the assessed alterations. This was based on the study by Zonato et al., which showed a statistically significant association between AHI and the presence of three or more of these same alterations.^3^ No statistical correlation was observed with respect to treatment success when comparing individuals who had these alterations with those who did not have them. There are no studies in the literature that have performed this type of correlation.

In the study by Marklund et al., the authors introduced new evidence in relation to the mechanism of action of MAD; according to them, nasal patency can be part of this mechanism, although they stated that more studies are required on the subject. Also in the same study, a correlation was observed between treatment failure and nasal obstruction, and this was more evident in women. Nasal obstruction assessment was performed subjectively in that study.^14^

Only one study assessed the influence of nasal resistance on the treatment of OSAS with MAD, in 38 patients selected for this treatment modality. Using rhinomanometry, Zeng et al. showed that patients considered as non-responders had higher nasal resistance in the sitting position than the responders, which was not observed when the same analysis was performed in the supine position. The authors also observed an increase in nasal resistance in non-responding patients when they were under the influence of MAD.^6^

In spite of the limitations of the present study due to the limited number of patients and the subjectivity of otorhinolaryngology physical examination, a significant association was observed between nasal alterations and treatment failure. These data confirm assumptions previously raised by other authors. It is known that nasal permeability increases during mandibular advancement in healthy patients, and this is one of the desired mechanisms of action with this device's use.^22^ Any anatomical alterations in this pathway (septal deviation, turbinate hypertrophy, nasal polyps) may interfere with the success of the applied therapy.^23^

Nasal obstruction can result in inadequate lip occlusion during sleep. Mouth breathing prevents a horizontal force from being applied to the jaw, an essential factor for the applicability of mandibular advancement, that limits the optimum performance of MAD.^24^

We believe that nasal obstruction may lead to an increase in the force performed during inspiration, which promotes upper airway collapse and, consequently, decreased efficiency of MAD in these patients. This hypothesis has been raised by Zeng et al., and the findings observed in this study further support it.^6^ Thus, we believe that an evaluation of UAs, especially of nasal obstructive factors, should be performed with caution during the initial evaluation of patients undergoing treatment for OSAS with MAD, as possible treatments of nasal alterations, such as those proposed for CPAP, may be adjuvants for the successful use of MAD.^25^

## Conflicts of interest

The authors declare no conflicts of interest.
